# Regional differences in general practitioners’ behaviours regarding influenza vaccination: a cross-sectional study

**DOI:** 10.1186/s12913-021-06177-x

**Published:** 2021-03-04

**Authors:** Jonathan Arlt, Kristina Flaegel, Katja Goetz, Jost Steinhaeuser

**Affiliations:** Institute of Family Medicine, University Hospital Schleswig-Holstein, Campus Luebeck, Ratzeburger Allee 160, 23538 Luebeck, Germany

**Keywords:** Influenza vaccination, General practitioners, Attitude, Vaccination behaviour, Regional differences

## Abstract

**Background:**

The World Health Organization recommends vaccination rates of 75% against seasonal influenza for patients over 65 years old. In the 2013/2014 season, the German vaccination rates ranged between 14 and 65%. This study aimed to compare the attitudes, personal characteristics and vaccination behaviours of general practitioners (GPs) in regions with high and low vaccination rates in Germany.

**Methods:**

In May 2016, a questionnaire was sent to 1594 GPs practising in 16 districts with the highest and the lowest vaccination rates in Western and Eastern Germany as described by the Central Research Institute of Ambulatory Health Care in Germany for the 2013/2014 season. Descriptive statistics and multiple regression analyses were computed to identify potential factors associated with high vaccination rates.

**Results:**

A total response rate of 32% (515/1594 participants) was observed in the study. GPs reported their attitudes towards vaccination in general and vaccination against influenza as mostly ‘very positive’ (80%, *n* = 352 and 65%, *n* = 288, respectively). GPs practising in regions with low vaccination rates reported their attitudes towards vaccinations in general (*p* = 0.004) and towards influenza vaccination (*p* = 0.001) more negatively than their colleagues from regions with high vaccination rates. Multiple logistic regression identified an increasing influence of year-dependent changing efficiency on GPs’ influenza rates as the strongest factor for predicting GPs from highly vaccinating regions (OR = 4.31 [1.12–16.60]), followed by the patient’s vaccination refusal despite GP advice due to already receiving a vaccination from another physician (OR = 3.20 [1.89–5.43]) and vaccination information gathering through medical colleagues (OR = 2.26 [1.19–4.29]).

**Conclusions:**

The results of this study suggest a correlation between GPs’ attitudes and regional vaccination rates. Beneath GPs’ individual attitudes, the regional attitude patterns of patients, colleagues and medical assistants surrounding those GPs seem decisive and should be integrated into future campaigns to increase vaccination rates at a regional level.

**Supplementary Information:**

The online version contains supplementary material available at 10.1186/s12913-021-06177-x.

## Background

Influenza is a potentially severe disease and is commonly underestimated by patients and professional caregivers, although it can cause a high number of deaths in epidemic seasons [1]. In 2009, the European Commission (EU) proposed the achievement for a 75% vaccination rate for those with an age ≥ 65 years that ended in 2015; this proposal was in reference to a suggestion from the World Health Organization (WHO) from 2003 [2, 3].

The German national immunisation schedule is developed by the Standing Committee on Vaccination (STIKO). Members of STIKO are unpaid, are experts in various fields and are appointed by the German Federal Ministry of Health for 3 years to act as an independent advisory group. By law, vaccination guidance from the federal states and the vaccination directive from the Federal Joint Committee are based on STIKO recommendations, which are not legally binding themselves [4]. According to the current recommendations, influenza vaccination is a standard vaccination for universal, yearly application in all adults aged 60 years old and older, an indicated vaccination for the risk groups of pregnant women (all pregnant women in the second trimester or in first trimester in cases of an underlying disease that increases their health risk) and persons of all ages with an increased health risk caused by an underlying disease, an indicated vaccination for residents of retirement or nursing homes and persons who might act as a potential source of infection for people with an increased health risk as well as a recommended vaccination due to an occupational risk with high contact with the public [5].

The German Federal Joint Committee decides about to which extent vaccinations belong to standard insurance benefits with regard to current STIKO recommendations [6, 7]. Thus, influenza vaccinations as standard and indicated vaccination or due to an occupational risk are free of charge for patients [8]. Physician’s reimbursement for the act of performing the vaccination is extra-budgetary and discretionary by health insurances [7]. Official numbers fluctuate around 8 Euros (7 British Pounds or 9 US Dollars) for a single vaccination [9, 10]. Next to the actual vaccination of the patient this reimbursement includes the need for consulting on vaccination benefits, possible adverse effects, complications and contraindications, recommended behaviour after getting vaccinated, start and duration of vaccination’s protection, the eventual need for more doses, and proper documentation [8].

In 2015, the Central Research Institute of Ambulatory Health Care in Germany (CRI) released a survey addressing the development of seasonal influenza vaccination rates in the 2013/2014 season. It was shown that vaccination rates ranged between low (13.5%) in Western Germany and high (64.9%) in Eastern Germany [11].

From 1949 to 1990, vaccination was voluntary in the Western Federal Republic of Germany (FRG), whereas it was compulsory in the Eastern German Democratic Republic (GDR). After the 1990s reunion of both states into Germany, voluntary vaccination paradigms continued. However, several studies showed that general practitioners (GPs) from the Eastern federal states of Germany continued vaccinating their patients more frequently than their Western colleagues did [11–13]. Studies showed that GPs’ recommendations have a distinctive impact on the decision of their patients to get vaccinated [12, 14–21].

Regional differences of vaccination coverage in France were attributed to GPs’ distrust in vaccine utility and information sources and GPs’ adherence to guidelines [22]. In addition to the important role of GPs, other sources of regional variations in vaccination coverage were identified. For instance, differences in budget reductions in public health spending were found to be associated with regionally varying measles, mumps and rubella coverage in Italy [23]. Moreover, variation in vaccination strategies due to local interests, socioeconomic conditions and public health priorities were also linked to regional differences in vaccination coverage [24].

To further investigate regional differences in vaccination rates, this study aimed to compare GPs’ attitudes, personal characteristics and vaccination behaviours as well as the role of external influences in regions with high and low vaccination rates in Germany.

## Methods

The study design was a cross-sectional study conducted via postal questionnaires.

### Questionnaire

Findings of a semi-structured literature research implicated the inclusion of items questioning sociodemographic data, attitudes, knowledge and continuing training, recommendation behaviour, vaccination practice, GP’s vaccination behaviour and employees’ vaccination status into the questionnaire [12, 14, 15, 17, 25–35]. Experiences of three GPs, one actively engaged in the STIKO, complemented these findings. The final questionnaire comprised 39 questions composed from a consensus process. As response formats, we used five-point Likert scales, categorical scales and free-text answers. The final questionnaire was checked on feasibility in a pre-test with two GPs. Since no corrections to the questionnaire had to be made, two GPs were considered enough for testing. The results of this pre-test were not included in the main analysis.

An English language version of the final questionnaire developed for this study is provided as Additional file 1.

### Study population

According to the Federal Registry of Physicians 2016, almost 55,000 physicians worked in primary care in Germany, including specialists in family medicine (64%), specialists in internal medicine (26%) and GPs without further specialisation (10%) [36]. Physicians in these three groups are summarized as GPs below.

In November 2015, the CRI published influenza vaccination rates for the 2013/2014 season using billing documents of all 17 German ‘Associations of Statutory Health Insurance Physicians’ (ASHIP) [11]. In Germany, ASHIP is responsible for mediation between remuneration requests of established physicians and the effective payment of the statutory health insurances, which approximately 86% of the German population belong to [37, 38].

Published data from the CRI were used for the acquisition of study participants by choosing the four districts with the highest vaccination rates and the four districts with the lowest vaccination rates in Eastern (former GDR) Germany. The same procedure was used for choosing districts in Western (former FRG) Germany, adding up to 16 districts in total. Vaccination rates in chosen districts ranged between 62.4 and 64.9% in Eastern regions, which had the highest vaccination rates, and between 42.2 and 44.8% in regions with the lowest vaccination rates. However, the highest vaccination rates in Western regions ranged between 46.6 and 48.0%, and the lowest vaccination rates ranged between 13.5 and 15.4%.

The regional ASHIP online registries were searched for the GPs working in the chosen 16 districts aiming for including all GPs into the survey reducing further self-selection bias. In total, the questionnaire including a postage-paid reply envelope was sent by regular mail to 1594 GPs in May 2016. The cover letter included information about the study’s reasons and benefits, the use of data and that participation is anonymous and takes about 10 min. No reminders were sent out. Because this was an exploratory study investigating GPs’ determinants of vaccination rates rather than proofing underlying concepts, no power calculation was determined.

### Statistical analysis

Data were analysed using IBM SPSS Statistics, version 24.0 (SPSS Inc., IBM Corporation, Armonk, NY, USA). For the descriptive analysis, categorical data were reported as frequencies and continuous data as means and standard deviations.

Subgroup analysis between GPs practising in regions with highest and lowest vaccination rates in Eastern and Western Germany was performed using chi-squared test for nominal-scaled data and Mann-Whitney U test for interval-scaled data. The same approach was used to evaluate differences between regions with highest vaccination rates in Western Germany and lowest vaccination rates in Eastern Germany, both showing similar vaccination rates.

Furthermore, a multiple logistic regression analysis was performed with the binary variable ‘vaccination rates’ (0 = low vaccination rates, 1 = high vaccination rates) as the outcome variable. Explanatory variables were chosen based on their thematic relevance and significance in subgroup analysis: GPs’ attitudes (towards vaccinations in general and influenza vaccination, distrust in pharmaceutical industry), GPs’ behaviour (influenza vaccination recommendation to employees, personal immunisation status, intention of getting vaccinated against influenza next season), external influences (adequate reimbursement, vaccine shortage, annually changing efficiency of the influenza vaccination, use of reminder systems, their own GP as influence for getting vaccinated against influenza for the first time, refusal of recommended vaccination by patients due to already being vaccinated by another physician), information resources (medical colleagues, scientifically trained sales representatives, continuing education courses/congresses, public media), influenza vaccination status of employees, and sociodemographic variables (age, sex). The Hosmer-Lemeshow test was used to evaluate the suitability of the logistic regression model [39]. Nagelkerke’s R squared explained the variance in the model [40]. Independent variables were entered into the forward stepwise regression model.

The lasso method was tested using Stata 15.0 (Stata Corp, College Station, TX, USA) in order to confirm the results of the multiple logistic regression analysis [41].

An alpha level of *p* < 0.05 was considered statistically significant.

### Ethics

Since this postal survey was planned and executed as collection of anonymous data, an informed written consent was not necessary to obtain from the participants. This approach was approved by the ethics committee of the University of Lübeck, Germany (File reference 16–077). Nevertheless, all GPs were fully informed in the cover letter about the consequences of participation, especially that the return of the questionnaires would result in data publication and will be seen as informed consent for participation.

## Results

### Sociodemographic data

In total, 453 questionnaires were returned (response rate of 28%). Since 10 participants refrained from indicating their specialty or were not GPs, 443 questionnaires were included in the analysis (28%). Of the participating GPs, 51% (*n* = 227) were male, were an average of 54 years old, and had practised in primary care for 19 years. Table 1 provides further details in comparison with the total population of German GPs according to the Federal Registry of Physicians in 2016 [36].

**Table 1:** Participants’ sociodemographic data (*n* = 505)

Sociodemographic data of responders (***n*** = 443)		Number	%	Total population of German GPs [36] n (%)
**Regional allocation of participants**	Regions with high vaccination rates	235	53.0	
	Regions with low vaccination rates	208	47.0	
**Sex**	Female	215	48.5	24,075 (43.9)
	Male	227	51.2	30,806 (56.1)
**Specialisation**	Specialist in family medicine	308	69.5	34,865 (63.5)
	Specialist in internal medicine (working in primary care)	111	25.1	14,853 (27.1)
	General practitioner without further specialisation	24	5.4	5163 (9.4)
**Practice Location**	Urban	223	50.3	
	Rural	212	47.9	
		**Mean**	**± SD**	**Total population of German GPs [36]** **Mean**
**Age**		54.0	± 9.4	55.1
**Years practising as primary care physician**		18.7	± 11.0	
**Patients visiting the practice in three months**		1136.3	± 421.3	

*SD* standard deviation

### Descriptive analysis

GPs reported their attitudes towards vaccination in general and vaccination against influenza mostly as ‘very positive’ (80%, *n* = 352 and 65%, *n* = 288, respectively). Knowledge about vaccinations in general and against influenza was stated to be optimal by 26% (*n* = 116) and 39% (*n* = 173), respectively. Information about vaccinations was gathered by medical journals (81%, *n* = 359), continuing education courses or conferences (81%, *n* = 357), scientific sales representatives (46%, *n* = 203), subject-specific web pages (38%, *n* = 170), medical colleagues (21%, *n* = 95) and public media (5%, *n* = 23). Five participants (1%) did not actively search for information.

Most participants considered influenza a dangerous disease (79%, *n* = 350), considered the influenza vaccination having few side effects (85%, *n* = 376) and stated from own experience that vaccinated persons would less frequently contract influenza (79%, *n* = 351). Eighty-five percent of participants (*n* = 377) followed STIKO recommendations. Financial reimbursement was considered adequate by 31% (*n* = 137) of participants. Fifteen participants (3%) would renounce vaccination recommendations due to missing financial incentives. More frequently, participants stated that vaccination recommendation renunciation was due to their patients’ contraindications (58%, *n* = 258), their own forgetfulness (54%, *n* = 240) and vaccine shortage (14%, *n* = 61). One-fifth of participants never renounced vaccination recommendations (*n* = 93).

**Fig. 1:**
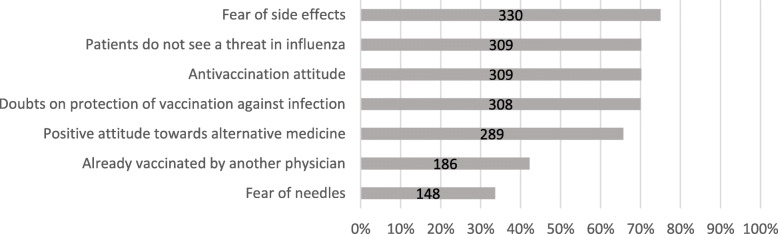
Patients’ reasons for refusing a recommended vaccination as experienced by GPs

Self-protection was stated as the main reason for getting vaccinated against influenza by 55% of participating GPs (*n* = 243), followed by protection of their patients (6%, *n* = 25) and protection of family and friends (5%, *n* = 23). Forty-three participants (10%) stated that they would not get vaccinated against influenza. Most GPs received their first influenza vaccination while practising in their own practice (41%, *n* = 182) and 8% of participating GPs (*n* = 36) received their first influenza vaccination during their medical studies. The biggest influence on their first influenza vaccination was nobody but themselves (40%, *n* = 178), followed by parents/family (18%, *n* = 79) and medical colleagues (17%, *n* = 73). One fifth of the participants (21%, *n* = 94) thought that physicians obtain too little information on vaccinations by public institutions, and 14% (*n* = 64) thought indications in vaccination recommendations to be unclear or confusing. Fifty-three participants (12%) did not think that unvaccinated GPs represent a danger of infection for their patients, whereas 79% (*n* = 350) thought that the patients’ decision for vaccination was substantially dependent on their recommendation as the patients’ GP. Participating GPs stated that the main reason for patients refusing a recommended vaccination is fear of side effects (75%). Other reasons are displayed in Fig. 1.

According to participating GPs, the vaccination status should mainly be checked during check-up examinations (97%, *n* = 428), at every patient contact (42%, *n* = 186), at visits specifically for that planned reason (41%, *n* = 180) or during home visits (36%, *n* = 161).

**Fig. 2:**
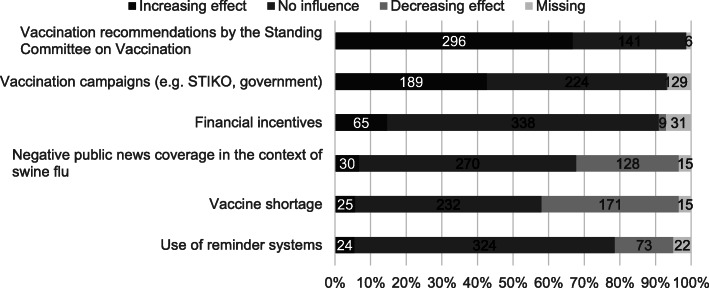
External factors influencing GPs’ vaccination rates from past years

Additionally, participating GPs rated several external factors influencing their vaccination rates from the past years. Whereas STIKO recommendations, vaccination campaigns and financial incentives were evaluated as having increasing effects, vaccine shortages and negative public news coverage in the context of the swine flu were stated to be the main reason for decreases in vaccinations. More details of the external factors for vaccination rates are displayed in Fig. 2.

### Subgroup analysis

Physicians practising in German regions with high vaccination rates did not differ significantly from participants in regions with low vaccination rates regarding sociodemographic data such as age (*p* = 0.144), sex (*p* = 0.482), specialisation (*p* = 0.154), years practised as primary care physician (*p* = 0.422), practice location (*p* = 0.086) and patients visiting in three months (*p* = 0.098).

Subgroup analysis revealed differences in items regarding GPs’ attitudes, GPs’ behaviour, external influences, information resources and influenza vaccination status of employees.

GPs practising in regions with low vaccination rates assessed their attitudes towards vaccinations in general (*p* = 0.004) and towards influenza vaccination (*p* = 0.001) more negatively than their colleagues from regions with high vaccination rates. Additionally, their personal vaccination status was more frequently incomplete (*p* = 0.020).

External influences (vaccine shortage, year-dependent changing effectiveness of the influenza vaccine, reminder system use) were more frequently stated as increasing and less frequently as decreasing on GPs’ vaccination rates by GPs in high vaccination rate regions (*p* = 0.008, *p* = 0.003. *p* = 0.027, respectively). Missing financial incentives (*p* = 0.040) and an inadequate financial reimbursement (*p* = 0.014) were more frequently perceived by those GPs from low vaccination rate regions than GPs working in high vaccination rate regions. Furthermore, the participants’ own GPs had a larger influence on their first influenza vaccination (*p* = 0.016).

For information gathering on vaccinations, GPs working in regions with high vaccination rates used information exchange more through medical education/congresses (*p* = 0.014) and with medical colleagues (*p* = 0.008) or scientific sales representatives *p* = 0.021) than their colleagues working in regions with low vaccination rates. GPs from low vaccination rate regions gathered information through public media more frequently (*p* = 0.025).

The employees of GPs were less frequently vaccinated in previous seasons (*p* = 0.007) and in the past five years (*p* = 0.014) while recommending the influenza vaccination less frequently (*p* < 0.001). More details are displayed in Table 2.

**Table 2:** Subgroup analysis between GPs practising in regions with high and low vaccination rates

Category	Items				Mean High^b^	Mean Low^c^	***p***-value
GPs’ attitudes	How do you assess your attitude towards vaccinations in general?^a^				4.83	4.70	0.004
	How do you assess your attitude towards influenza vaccination?^a^				4.66	4.37	0.001
				**Agree%** **(N)**	**Neutral%** **(N)**	**Disagree%** **(N)**	**p-value**
	The influenza vaccination only benefits the pharmaceutical industry.		**High**	0.4 (1)	8.9 (21)	90.6 (213)	0.002
			**Low**	2.4 (5)	18.4 (38)	79.1 (163)	
GPs’behaviour				**Yes%** **(N)**	**Maybe%** **(N)**	**No%** **(N)**	**p-value**
	Will you get vaccinated against influenza next season?		**High**	87.6 (205)	7.7 (18)	4.7 (11)	0.024
			**Low**	77.9 (162)	13.0 (27)	9.1 (19)	
					**Yes%** **(N)**	**No%** **(N)**	**p-value**
	Is your personal vaccination status complete?		**High**		96.6 (226)	3.4 (8)	0.020
			**Low**		91.3 (190)	8.7 (18)	
	I have never been vaccinated against influenza.		**High**		1.7 (4)	98.3 (227)	0.001
			**Low**		8.8 (18)	91.2 (187)	
	Do you recommend the influenza vaccination to your employees?		**High**		98.7 (231)	1.3 (3)	< 0.001
			**Low**		91.2 (187)	8.8 (18)	
				**Yes%** **(N)**	**Partly/partly%** **(N)**	**No%** **(N)**	**p-value**
External influences	I perceive financial reimbursement from vaccinations as adequate.		**High**	34.5 (79)	45.9 (105)	19.7 (45)	0.014
			**Low**	28.4 (58)	39.7 (81)	31.9 (65)	
				**Increasing%** **(N)**	**No influence%** **(N)**	**Decreasing** **(N)**	**p-value**
	Influence of vaccine shortage on GP’s own vaccination rates		**High**	7.5 (17)	59.2 (135)	33.3 (76)	0.008
			**Low**	4.0 (8)	48.5 (97)	47.5 (95)	
	Influence of year-dependent changing effectiveness of the influenza vaccine on GP’s own vaccination rates		**High**	6.6 (15)	81.9 (185)	11.5 (26)	0.003
			**Low**	4.6 (9)	71.3 (139)	24.1 (47)	
	Influence of reminder system use on GP’s own vaccination rates		**High**	44.6 (87)	54.9 (107)	0.5 (1)	0.027
			**Low**	31.3 (55)	67.6 (119)	1.1 (2)	
					**Yes%** **(N)**	**No%** **(N)**	**p-value**
	I renounce a vaccination recommendation to (part of) my patients due to missing financial incentives.		**High**		1.7 (4)	98.3 (227)	0.040
			**Low**		5.3 (11)	94.7 (196)	
	Patients reject a recommended vaccination on the ground that another physician has already vaccinated them.		**High**		52.6 (123)	47.4 (111)	< 0.001
			**Low**		30.6 (63)	69.4 (143)	
	My GP had a big influence on my first influenza vaccination.		**High**		12.7 (29)	87.3 (200)	0.016
			**Low**		21.4 (42)	78.6 (154)	
Information resources	I gather information on vaccinations through medical colleagues.		**High**		26.4 (62)	73.6 (173)	0.008
			**Low**		15.9 (33)	84.1 (174)	
	I gather information on vaccinations through scientific sales representatives.		**High**		51.1 (120)	48.9 (115)	0.021
			**Low**		40.1 (83)	59.9 (124)	
	I gather information on vaccinations through continuing medical education/congresses.		**High**		85.1 (200)	14.9 (35)	0.014
			**Low**		75.8 (157)	24.2 (50)	
	I gather information on vaccinations through public media (radio, TV, websites without review).		**High**		3.0 (7)	97.0 (228)	0.025
			**Low**		7.7 (16)	92.3 (191)	
				**Yes%** **(N)**	**Partly/partly%** **(N)**	**No%** **(N)**	**p-value**
Employees’ vaccination status	Were your employees vaccinated against influenza past season?		**High**	47.2 (119)	48.1 (112)	4.7 (11)	0.007
			**Low**	38.8 (80)	48.5 (100)	12.6 (26)	
	Have your employees been vaccinated against influenza in the past five years?		**High**	38.8 (90)	57.8 (134)	3.4 (8)	0.014
			**Low**	31.5 (64)	58.6 (119)	9.9 (20)	

^a^assessed on a five-point Likert scale (1 = “very negative”, 5 = “very positive”)

^**b**^GPs practising in regions with high vaccination rates

^c^GPs practising in regions with low vaccination rates

Subgroup analysis between regions with highest vaccination rates in Western Germany and lowest vaccination rates in Eastern Germany revealed differences in items regarding knowledge, GPs vaccination behaviour, external influences - amongst others the vaccination refusal by patients - and vaccination status of employees. GPs in regions with the lowest vaccination rates in Eastern Germany were more often female (*p* = 0.003), stated a higher knowledge level about vaccines in general (*p* = 0.031), more of their employees being vaccinated against influenza in the last season (*p* = 0.004) and in the past five years (*p* < 0.001), more often their GP as influence to their decision on being vaccinated against influenza for the first time (p = 0.004), more often missing financial incentives for vaccinations (*p* = 0.016) and an increased offering of influenza vaccination in university as a student (*p* = 0.002) than their colleagues in regions with highest vaccination rates in Western Germany. On the contrary, GPs in regions with highest vaccination rates in Western Germany were practising more often in rural areas (*p* < 0.001), stated to vaccinate more often patients with acute infections without fever (p < 0.001), patients allergic to egg white (*p* = 0.043), immunosuppressed patients (*p* = 0.010), more often an vaccination rate increasing effect of reminder systems (*p* < 0.001) and vaccination campaigns (*p* = 0.035), more often patients refusing their advice due to a positive attitude towards alternative medicine (*p* = 0.018), fear of side effects (p = 0.010), fear of needles (*p* = 0.032) or a critical attitude towards vaccines (p < 0.001), and more often that nobody but them influenced their decision to get vaccinated against influenza for the first time (*p* = 0.004) than their colleagues in Eastern Germany. GPs in Western Germany agreed less often that physicians’ attitudes were already shaped before being a physician (*p* = 0.008).

### Multiple logistic regression

The binary regression model identifying associated factors with GPs practising in regions with high vaccination rates showed seven factors with a Nagelkerke R^2^ of 0.245 (Hosmer-Lemeshow test *p* = 0.394) and is presented in Table 3. The strongest factors belonged to external influences and information resources: an increasing influence of year-dependent changing efficiency on their own influenza rates (OR = 4.31 [1.12–16.60]), the patient’s vaccination refusal despite GP advice due to already performed vaccination by another physician (OR = 3.20 [1.89–5.43]) and vaccination information gathering through medical colleagues (OR = 2.26 [1.19–4.29]). Negative factors - as well belonging to information resources and external influences - were information gathering through public media (OR = 0.16 [0.05–0.53]) and the GP as a significant influence on participating GPs’ first influenza vaccination (OR = 0.41 [0.21–0.81]).

**Table 3:** Factors associated with GPs practising in regions with high vaccination rates: a multiple logistical regression model

Category	Variables	OR	95% CI		p-value
			lower value	upper value	
External influences	Influence of year-dependent changing efficiency of influenza vaccination on own vaccination rates				
	Decreasing				0.022
	No influence	2.79	1.29	6.05	0.009
	Increasing	4.31	1.12	16.60	0.034
External influences	Refusal of vaccination by patient despite GP advice due to already performed vaccination by another physician	3.20	1.89	5.43	< 0.001
Information resources	Information gathering regarding vaccinations through medical colleagues	2.26	1.19	4.29	0.012
External influences	Influence of vaccine shortage on own vaccination rates				
	Decreasing				0.045
	No influence	1.88	1.08	3.26	0.025
	Increasing	2.95	0.80	10.86	0.104
External influences	Influence of reminder system use on own vaccination rates				
	Decreasing				0.004
	No influence	0.40	0.01	11.31	0.589
	Increasing	1.01	0.35	28.93	0.994
External influences	GP as big influence on first influenza vaccination	0.41	0.21	0.81	0.010
Information resources	Information gathering regarding vaccinations through public media (radio, TV, websites without review)	0.16	0.05	0.53	0.002

*OR* odds ratio, *CI* confidence interval

The lasso method confirmed the results of the multiple logistic regression; the estimators did not differ from the used multiple logistic regression.

## Discussion

To our knowledge, this is the first study that compares GPs’ attitudes and vaccination behaviours towards influenza vaccination in regions with high and low vaccination rates in one country. GPs’ attitudes and behaviour as well as external influences and information resources have been identified as factors associated with regional differences in vaccination coverage. Thus, a more positive perception of the year-dependent changing efficiency of the influenza vaccination, of vaccine shortage or reminder systems and the patients’ refusal to get vaccinated due to an already performed vaccination by another physician as well as medical colleagues as information resources are factors linked to regions with high vaccination rates.

Generally, participating GPs have an overall positive attitude towards influenza vaccination, consistent with pre-existent studies [14], whereas an optimal state of knowledge still seems expendable. Since medical journals, continuing education courses and medical congresses are the most important media for information gathering on vaccinations, future campaigns should set their focus there.

The ability to discuss side effects correctly with patients seemed to be especially important, as patients from physicians’ experiences refuse vaccinations mostly because of their fear of side effects. A study showed that nurse practitioners use more time in discussing side effects with patients than GPs, possibly leading to higher levels of patients’ satisfaction with care [42]. It has been shown that training physicians in communication results in substantially higher patient adherence [43]. Unfortunately, communication training is not explicitly part of postgraduate training in Germany [44] and communication training’s extent in medical school is not determined but the achievement of communicative competencies is recommended [45, 46].

Information by public institutions and other providers must be clearly comprehensible. Therefore, everyday users need to be involved in drafting recommendations or at least testing them. This strategy might also enhance the percentage of GPs following official STIKO recommendations; currently, 85% of GPs follow the STIKO recommendations. This number seems insufficient considering that 75% of patients over 65 years should be immunized according to the WHO, and GPs forgetfulness was stated as the main reason for renouncing vaccination recommendations. Thus, neglectful or reluctant recommendation behaviour might be a determining factor of low vaccination rates.

Only 42% of participating GPs thought every patient contact was appropriate for checking the patient’s immunisation status, and 97% thought check-up examinations to be appropriate. In Germany, regular check-up examinations are performed every two years starting at age 35 and are voluntarily on patients’ request [47]. This missing routine in checking the patient’s immunisation status might lead to GP forgetfulness in recommending vaccinations. The U.S. Standards for Adult Immunisation Practice stipulated to assess adult patients’ vaccination status at every visit [48]; in addition, the German STIKO states that every patient contact should be used for assessing patient’s immunisation status [49]. Patient and physician reminder or recall systems internationally showed effectiveness in increasing patients’ vaccination rates [49–51]. Notably, our study showed that reminder systems – not specified whether patient or physician reminders - were more often rated as decreasing vaccination rates rather than increasing vaccination rates. Recent results from three German intervention studies with the aim of increasing influenza vaccination rates in patients with chronic renal disease implied that only vaccination reminders addressed directly to the patient and not to the physician had a positive effect on patients’ vaccination rates [52]. A decreasing effect was not reported. Hence, this finding would need further attention in future research in order to evaluate this result’s reliability.

Participating GPs practising in regions with low vaccination rates stated their attitude to be slightly more negative than their colleagues from highly vaccinating regions, whereas self-rated knowledge showed no significant differences. Therefore, interventions that are able to change attitudes might be more appropriate to increase vaccination rates than pure knowledge provision. Behaviour change frameworks have been shown to predict vaccination behaviour of health care workers and are referred to as a “promising tool” to increase the influenza vaccination uptake in health care workers [53].

Moreover, their GPs have a larger influence on participating GPs’ first influenza vaccination in regions with lower vaccination rates. Thus, it is not the families that make participating GPs get vaccinated. This finding supports the idea that attitude, which is formed early in life, highly influences physicians’ vaccination rates.

Financial reimbursement was seen as adequate by only 31% of all participants. In subgroup analysis, it was shown that missing financial incentives and inadequate financial reimbursement were stated by GPs practising in regions with lower vaccination rates. In addition, other external influences (vaccine shortage, year-dependent changing effectiveness of the influenza vaccine, reminder system use) were more often seen as decreasing factors by those GPs. Supposedly, these external influences might be used as excuses by GPs for not vaccinating to the extent that is officially recommended. However, payment to physicians was identified as a successful intervention to increase influenza vaccination rates of those 60 years old and older in the community [51].

Participants practising in regions with high vaccination rates showed a higher tendency towards information gathering by scientific sales representatives rather than public media. However, the openness to scientific sales representatives must be seen critically as former studies showed that GPs provided with information by scientific sales representatives might prescribe more frequently, generate higher costs or resort to qualitatively lower prescriptions [54, 55]. In a different study, half of participating German GPs stated feeling not informed correctly by pharmaceutical sales representatives, and three-quarters felt an effort of influencing their prescription behaviour [56].

According to participating GPs’ statements, medical assistants in regions with low vaccination rates are more seldomly vaccinated than medical assistants in regions with high vaccination rates. Medical assistants’ low vaccination rates match pre-studies [14, 30, 57]. Ascertained reasons were scepticism about side effects and infections caused by a vaccination [30]. Continuing education of medical assistants concerning vaccination needs to be improved, especially since previous vaccination campaigns tended to be less powerful for health care workers [57].

The perceived increasing influence on participants’ vaccination rates due to the changing efficiency of the influenza vaccination was identified as strongest predictor for GPs practising in regions with high vaccination rates. They might see the year-dependent changing efficiency as reason why only a regular, yearly vaccination seems reasonable for their patients. At the same time the concerns about vaccines efficiency might discourage GPs from vaccinating patients in regions with low vaccination rates. This finding needs further attention in future studies since the missing documentation of actual vaccination strategies by participating GPs in this study precludes confirmation of this assumption. Clearly, mismatches between current circulating viruses and used vaccine strains reduce vaccine efficacy [58, 59]. However, influenza vaccination still is described as most effective disease prevention intervention [59].

GPs practising in regions with high vaccination rates stated more often with statistical significance that patients would reject their vaccination recommendation because they had already been vaccinated by another physician, being the second most important factor in multiple logistic regression analysis. This finding implies that those GPs are generally working in a vaccine-friendly region. Hence, measurements reaching individual GPs might not be enough to change vaccination rates on a regional level, especially when GPs rely on their medical colleagues to gather information about vaccination. Beneath emphasizing the importance of vaccination recommendations to practising GPs, campaigns should effectively increase the focus on regionally differing conditions and the patients’ attitudes towards vaccination. According to the results of the subgroup analysis comparing regions with the same vaccination rates in Eastern and Western Germany, patients’ attitudes play a major role whether a vaccination actually takes place in Western Germany.

### Strengths and limitations

With a response rate of 28%, this study achieved a response rate comparable to other studies in health services research in Germany [60]. Postal surveys are prone to higher percentages of missing responses. Nevertheless, they are simple, fast and inexpensive to execute like cross-sectional surveys in general [61].

In total, 28% of all contacted GPs are represented in the study’s results, accounting for 0.8% of all German GPs in 2016 (*n* = 54,605) [36]. The population-level comparison as valuable evaluation of non-response bias [62] showed that participants’ sociodemographic data were equivalent to the total population of German GPs according to the Federal Registry of Physicians. German GPs have an average age of 55 years, and 44% are female [36]. The distribution of specialisations is comparable to the study population. However, other known approaches eventually more appropriate to assess non-response bias were not included in the study design. Thus, the real of determinants of GPs’ behaviour regarding influenza vaccination in the sample might differ from the population due to non-response bias.

A random sampling method could not be used, as for this cross-sectional study we invited all GPs of the districts with the lowest and highest vaccination rates in Eastern and Western Germany according to published influenza vaccination rates for the 2013/2014 season by the CRI. Therefore, a self-selection bias might be present. Hence, conclusions about the whole population are severely limited even when sociodemographic data of responders and population are similar.

Assuming that participating GPs from regions with high or low vaccination rates are those GPs that actually vaccinate more or less often, respectively, is a further limitation.

This was a cross-sectional study, and thus, we must be cautious to derive causal links from these findings. Since social desirability and recall bias are present in cross-sectional surveys, answers regarding the frequency of own and practice assistants’ and patients’ vaccinations as well as recommendation behaviour might be presented by the participants more positively than actually happening. Immunisation certificates or other medical documentation were not obtained to proof information given by responders.

Finally, only those determinants of GPs’ behaviours were assessed that were identified through our preceding literature search. There might be others, which were not presented here.

## Conclusions

The results of this study suggest a correlation between GPs’ attitudes and regional vaccination rates. Beneath GPs’ individual attitudes, the regional attitude patterns of patients, colleagues and medical assistants surrounding those GPs seem decisive and should be integrated into future campaigns to increase vaccination rates on a regional level.

## Supplementary Information

**Additional file 1.** Questionnaire “General Practitioners’ Attitudes Regarding Vaccinations”. English language version of the final questionnaire developed for this study.

## Data Availability

The datasets used and analysed during the current study are available from the corresponding author on reasonable request.

## Abbreviations

GPs: General practitioners
EU: European Commission
WHO: World Health Organization
STIKO: Standing Committee on Vaccination (Germany)
CRI: Central Research Institute of Ambulatory Health Care (Germany)
FRG: Federal Republic of Germany
GDR: German Democratic Republic
ASHIP: Association of Statutory Health Insurance Physicians
