# Supplementary material for: Regional differences in general practitioners’ behaviours regarding influenza vaccination: a cross-sectional study
Source: BMC Health Serv Res. 2021 Mar 4;21:197. doi: 10.1186/s12913-021-06177-x (PMC7934451; doi:10.1186/s12913-021-06177-x)
Supplement: Supplementary file 1 — Additional file 1. Questionnaire “General Practitioners’ Attitudes Regarding Vaccinations”. English language version of the final questionnaire developed for this study. [file 12913_2021_6177_MOESM1_ESM.pdf]

General Practitioners' Attitudes Regarding Vaccinations

exemplified by the standard vaccinations against influenza and invasive pneumococcal disease

Dear colleague,

Vaccinations belong to the most effective preventive measures that can be performed by us as physicians. In order to better understand variabilities in vaccination rates throughout Germany, we would like to invite you to inform us about your attitudes towards vaccinations against influenza and invasive pneumococcal disease in this questionnaire. This survey is conducted anonymously. With the return of the questionnaire you consent to the scientific analysis of your responses by the Institute of Family Medicine of the University Hospital Schleswig-Holstein, Campus Lübeck. Your data will be treated strictly confidentially. There will be no circulation of your data to a third party.

Please consider the following instructions while filling in the questionnaire:

- Please only use a blue or black biro.
- Please answer free text in printed letters.
- Please answer all questions by clearly checking the boxes next to your answer: ☒
- If you have to correct yourself, please fill in the box completely: ■

  and check the box with your answer again: ☒

Thank you for your participation!

---

1. How do you assess your attitude towards vaccinations…

	very positive				very negative
	1	2	3	4	5
…in general?	☐	☐	☐	☐	☐
…against influenza?	☐	☐	☐	☐	☐
…against invasive pneumococcal disease?	☐	☐	☐	☐	☐

2. How do you assess your knowledge about vaccinations…

	optimal				unsatis-factory
	1	2	3	4	5
…in general?	☐	☐	☐	☐	☐
…against influenza?	☐	☐	☐	☐	☐
…against invasive pneumococcal disease?	☐	☐	☐	☐	☐

3. **Which of the following statements apply to your personal attitude towards vaccinations?**

	Yes	Partly/partly	No
I follow recommendations by the Standing Committee on Vaccination (STIKO).	☐	☐	☐
I perceive financial reimbursement from vaccinations as adequate.	☐	☐	☐
I consider influenza a dangerous disease.	☐	☐	☐
From my own experience vaccinated persons less frequently contract influenza.	☐	☐	☐
I consider the influenza vaccine having few side effects.	☐	☐	☐
I consider pneumococcal infections dangerous.	☐	☐	☐
From my own experience vaccinated persons less frequently contract an infection with pneumococcus.	☐	☐	☐
I consider the pneumoccocal vaccine having few side effects.	☐	☐	☐

4. **Which of the following is the <u>most important</u> reason for you to get vaccinated against influenza?**

☐ Protection of family/friends
☐ Self-protection (against infection/sick leave)
☐ Protection of my patients
☐ Other, namely: ____________________
☐ I do not get vaccinated against influenza.

5. **Do you regularly vaccinate your patients in the following situations…?**

	Yes	Partly/partly	No
…acute non-febrile infections *(e.g. pharyngitis)*?	☐	☐	☐
…acute febrile infections *(e.g. bronchitis)*?	☐	☐	☐
…allergy to egg whites *(e.g. with alternative vaccine)*?	☐	☐	☐
…immunosuppression *(e.g. caused by disease or medication)*?	☐	☐	☐

6. **Has your attitude towards the influenza vaccination changed in the past few years?**

☐ Yes, namely: ____________________
____
☐ No.

7. **Has your attitude towards the vaccination against invasive pneumococcal disease changed in the past few years?**

☐ Yes, namely: ____________________
____
☐ No.

8. **How did the following factors influence your patient vaccination rates from the past years?**

	Increasing influence	No influence	Decreasing influence	Not known
Vaccination recommendations by the STIKO	☐	☐	☐	☐
Vaccination campaigns (e.g. STIKO, government)	☐	☐	☐	☐
Financial incentives	☐	☐	☐	☐
Patient being a member of the statutory health insurance	☐	☐	☐	☐
Patient being a member of the private health insurance	☐	☐	☐	☐
Patient's participation in the programme „family doctor coordinated care" (HzV)	☐	☐	☐	☐
Vaccine shortage	☐	☐	☐	☐
Negative public news coverage in the context of swine flu	☐	☐	☐	☐
Year-dependent changing effectiveness of the influenza vaccine	☐	☐	☐	☐
Benefits of the German College of General Practitioners and Family Physicians (DEGAM)	☐	☐	☐	☐
Use of reminder systems	☐	☐	☐	☐

9. **What is your level of agreement regarding the following statements?**

	Agree	Neutral	Disagree
Physicians obtain too little information on vaccinations by public institutions.	☐	☐	☐
I think that indications in vaccination recommendations are unclear or confusing.	☐	☐	☐
The patient's decision for vaccination is substantially dependent on my recommendation as his/her general practitioner.	☐	☐	☐
The attitude of the physician towards vaccinations has been shaped even before starting the medical profession.	☐	☐	☐
Unvaccinated physicians represent a danger of infection for their patients.	☐	☐	☐
The influenza vaccination only benefits the pharmaceutical industry.	☐	☐	☐
The vaccination against invasive pneumococcal disease only benefits the pharmaceutical industry.	☐	☐	☐

10. Do you advocate for mandatory vaccinations against influenza?

- ☐ Yes, mandatory vaccinations for all adults in general.
- ☐ Yes, only for health professionals.
- ☐ No.

11. Do you recommend the inlfuenza vaccination to your employees? ☐ Yes. ☐ No.

12. Were your employees vaccinated or have your employees been vaccinated against influenza…?

	Yes, all of them	Partly/ partly	No
…past season	☐	☐	☐
…in the past five years	☐	☐	☐

13. What percentage of patients of the following groups do you usually recommend the vaccination against influenza to?

…patients older than 60 years ⎣__⎦ %

...patients with chronic diseases ⎣__⎦ %

…pregnant patients ⎣__⎦ %

14. <u>Of those</u>, what percentage actually got vaccinated against influenza?

…patients older than 60 years ⎣__⎦ %

…patients with chronic diseases ⎣__⎦ %

…pregnant patients ⎣__⎦ %

15. What percentage of patients of the following groups do you usually recommend the vaccination against invasive pneumococcal disease to?

…patients older than 60 years % ⎣__⎦

...patients with chronic diseases % ⎣__⎦

16. <u>Of those</u>, what percentage actually got vaccinated against invasive pneumococcal disease?

…patients older than 60 years ⎣__⎦ %

…patients with chronic diseases ⎣__⎦ %

17. If you renounced vaccination recommendation for (part of) your patients, why would you renounce vaccination recommendation? *Multiple answers possible.*

- ☐ The vaccine is in (very) short supply.
- ☐ There are contraindications during the patient encounter.
- ☐ I occasionally forget vaccination recommendations.
- ☐ Adequate financial incentives are missing.
- ☐ Others, namely: ____________________
- ☐ I never renounce vaccination recommendations.

18. What is the reason why patients reject a vaccination that was recommended by you?
Multiple answers possible.

- ☐ Positive attitude towards alternative medicine
- ☐ Patients do not see a threat in influenza
- ☐ Patients do not see a threat in invasive pneumoccocal disease
- ☐ Fear of side effects
- ☐ Fear of needles
- ☐ Doubts on protection of vaccination against infection
- ☐ Already vaccinated by another physician
- ☐ Antivaccination attitude
- ☐ Others, namely: ____________________

19. How do you gather information about vaccinations? *Multiple answers possible.*

- ☐ Medical colleagues
- ☐ Scientific sales representatives
- ☐ Continuing education courses/conferences
- ☐ Medical journals
- ☐ Subject-specific web pages (Pubmed, electronic journals, STIKO)
- ☐ Public media (radio, TV, web pages without review)
- ☐ I do not actively search for information.

20. Do you consider the financial reimbursement of vaccinations in general adequate?

In the statutory health insurance	☐ Yes.	☐ No.
In the private health insurance	☐ Yes.	☐ No.

21. Ideally, when should the vaccination status be checked?

During check-up examinations (e.g. check-up starting at age 35)	☐ Yes.	☐ No.
During home visits	☐ Yes.	☐ No.
At every patient contact	☐ Yes.	☐ No.
At visits specifically planned for that matter	☐ Yes.	☐ No.

Others, namely: ____________________

22. What percentage of vaccines used by you against influenza are...

...trivalent vaccines |_|_|_| ?

...nasal vaccines |_|_|_| ?

...quadrivalent vaccines |_|_|_| ?

23. What percentage of vaccines used by you against invasive pneumococcal disease are...

...conjugated vaccines |_|_|_| ?

...polysaccharide vaccines |_|_|_| ?

Practice profile and personal information

24. Is your personal vaccination status complete? ☐ Yes. ☐ No.

25. Do you currently have a vaccination card? ☐ Yes. ☐ No.

26. How often have you been vaccinated in the past ten years? ______ times

27. Will you get vaccinated against influenza next season? ☐ Yes. ☐ Maybe. ☐ No.

28. Was influenza vaccination offered at your university when you were a student?
☐ Yes. ☐ No.

29. When did you receive your first influenza vaccination?

- ☐ Before studying medicine
- ☐ While studying medicine
- ☐ While practising in hospital
- ☐ While practising in practice
- ☐ I have never received an influenza vaccination.

30. Who had the biggest influence on your first influenza vaccination? *Multiple answers possible.*

- ☐ Parents/family
- ☐ General practitioner
- ☐ Professors/lecturers at university
- ☐ Company physician
- ☐ Medical colleagues
- ☐ Nobody but myself
- ☐ Health professionals in the hospital/practice
- ☐ Others, namely:____

31. How old are you? ___________ years

32. You are…. ☐ female. ☐ male.

33. At which medical school did you study? ______________________________________

34. What is your specialisation?

☐ Specialist in family medicine ☐ Specialist in internal medicine
☐ General practitioner without further specialisation ☐ Other:____________________

35. For how long have you been practising as primary care physician? _______ years

36. How many patients do you see in three months? __________ [number]

37. Where is your practice located? ☐ In town ☐ In the countryside

38. Do you participate in the programme „family doctor coordinated care“ (HzV)?

☐ Yes, _____ % of patients are registered.
☐ No, I refuse this programme.
☐ No, this programme is not offered in my federal state.
☐ I do not know this programme.

39. Have you found an important aspect missing in this questionnaire? If yes, which one?

____

____

____

Thank you for your participation!
